# Identifying Potential Child Abuse through Oral Examination

**DOI:** 10.1155/2017/6943954

**Published:** 2017-04-02

**Authors:** Jillian N. Printz, Aaron Baker, Michele Carr

**Affiliations:** ^1^College of Medicine, The Pennsylvania State University, Hershey, PA, USA; ^2^Department of Surgery, College of Medicine, The Pennsylvania State University, Hershey, PA, USA

## Abstract

Limited reports of oropharyngeal trauma exist in the literature even though this type of injury is extremely common in pediatric populations. There are no widely agreed upon diagnostic and management tools for such injuries in abuse cases, emphasizing the importance of reporting rare cases of orofacial trauma. This case report of a soft palate laceration demonstrates an instance of initially unrecognized potential child abuse. We aim to clarify understanding of such injuries. Furthermore, the report highlights the need for recognition of oral signs of child abuse in order to promote early detection, reporting, and appropriate management.

## 1. Introduction

The oropharynx is bounded by the soft palate and uvula superiorly, tongue inferiorly, dentition and buccal mucosa anterolaterally, palatine tonsils and tonsillar pillars posterolaterally, and the posterior pharyngeal wall. Oropharyngeal impalement injury accounts for 1-2% of all pediatric trauma [1]. Intraoral and facial trauma are reported in up to 49% of infants and 38% of toddlers who are physically abused [2]. Proposed mechanisms of injury include forced feeding, gagging, or direct blow to the area [2]. Children under age of 2 are at the highest risk of oral and facial injuries due to abuse, including retinal hemorrhage, facial bruises and burns, fractured or avulsed teeth, fractures of the mandible or maxilla, and lacerations to the lips, frenula, and tongue [3].

While the vast majority of oral and oropharyngeal trauma cases resolve spontaneously, laceration may require surgical repair. We recently encountered a patient with a unique pattern of injury to the soft palate. By reporting this case, we aim to broaden knowledge of recognition and management of palatal laceration and emphasize the importance of recognizing the oral signs of child abuse for all medical professionals.

## 2. Case

**Figure 1:**
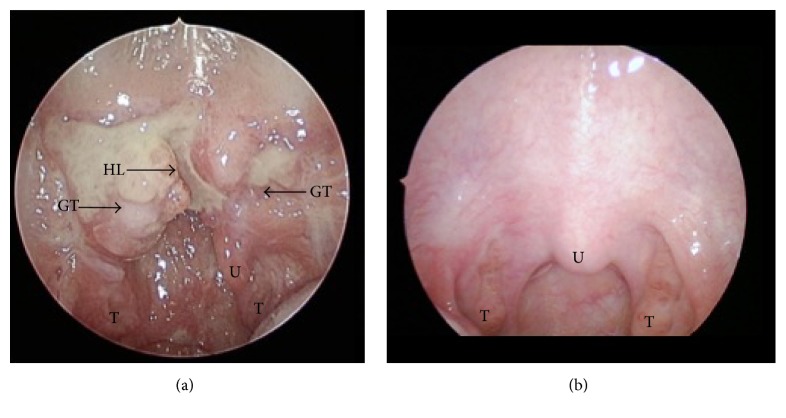
(a) Healing granulation tissue of the soft palate with a deviated uvula; (b) normal palate for comparison; U = uvula; T = tonsil; HL = healing laceration; GT = granulation tissue.

A 14-month-old female presented to the Emergency Department with a history of sore throat, irritability, and restlessness. The patient's mother reported that she was first diagnosed with a right-sided otitis media for this complaint 3 weeks prior to this presentation and was treated with amoxicillin. Six days later, after her mother noticed a “white spot” in the back of her throat, the child was reassessed and treated with nystatin for thrush. The amoxicillin was switched to cefdinir for suspected pharyngitis; however, the symptoms persisted. Three days later, the child was brought to her pediatrician, who started the patient on fluconazole. The patient's oral intake was limited due to suspected odynophagia, to the point of no oral intake while under the care of her babysitter, with an associated 1 lb (4.34%) weight loss over 2 weeks. Mother denied any known injuries. On physical examination, the emergency medicine physician noted a creamy white colored plaque on the soft palate, tonsillar hypertrophy with erythema, and yellow mucoid discharge. A neck X-ray was performed and revealed thickened prevertebral soft tissues with narrowing of the airway at the level of C4. Subsequently, a CT scan with contrast was ordered due to a concern for retropharyngeal abscess. The scan showed prominence of the adenoid and tonsils with no evidence of retropharyngeal abscess. One day after arrival to the Emergency Department, the emergency medicine physician consulted the pediatrics team, and the pediatrics team admitted the patient to the hospital. Physical examination by the pediatric team reported an erythematous oropharynx with 2-3+ tonsillar hypertrophy and bilateral exudates. The patient was started on Ampicillin/Sulbactam. The next day, at 3 weeks after onset of symptoms, the pediatrics team consulted otolaryngology. On physical examination, the otolaryngology team noticed an extensive, partially healed soft palate laceration. A complete oral examination was not possible, as the patient was not compliant. It was then recommended that she undergoes examination under anesthesia. This exam showed that the patient's uvula was deviated toward the left (Figure 1). The left soft palate appeared irregular, consistent with a healing laceration. There was a noted soft palate cleft, which was partially mucosalized on the right, adjacent to the uvula. Lateral to the cleft, there was a 1.5 cm × 2 cm growth with an irregular surface, suspicious for granulation tissue. The remainder of the oral and oropharyngeal tissues appeared normal. A small piece of the right palate mass was sent for histopathology, which confirmed healing granulation tissue, consistent with an injury likely some weeks prior.

The state of the tissue at the time of presentation did not allow for repair. Due to concern for possible abuse, a report was filed with ChildLine.

The patient was followed closely in the clinic. By 3 months after injury, she had bilateral middle ear effusions, weight loss of 3.3 pounds, and persistent poor feeding. There was no evidence of nasopharyngeal regurgitation of feeds clinically or on a swallowing study. Oral examination reveals a scar on her soft palate that has remodeled. There is no evidence of the posterior palatal notch that she had previously. No other signs or physical exam findings of abuse were noted.

## 3. Discussion

The four muscles of the soft palate (palatoglossus, palatopharyngeus, tensor veli palatini, and levator veli palatini) aid in swallowing and are critical in opening of the Eustachian tubes; thus, injury to the palatal region can impair these functions. The palatoglossus muscle elevates the tongue and depresses the soft palate. The palatopharyngeus muscle elevates the pharynx to pull it over a bolus a food, helping to prevent regurgitation of food into the nasopharynx. During swallowing, constriction of the tensor veli palatini tenses the soft palate and constriction of the levator veli palatini elevates the soft palate, opening the Eustachian tube orifice along with the tensor tympani and the salpingopharyngeus muscles. The superior attachment of salpingopharyngeus muscle is to the cartilage of the Eustachian tube. In the case presented, damage to the palate, especially the tensor veli palatini muscle, can cause impaired opening of the Eustachian tube resulting in negative middle ear pressure and effusion [4]. Long term sequelae of Eustachian tube dysfunction include otalgia, hearing loss, tinnitus, and chronic otitis media [4].

This case represents one of very few reports on oropharyngeal laceration. Previous case reports of both abuse and nonabuse cases document lesions of the hard palate, laceration of the pharyngeal wall, and injury to the oral mucosa, upper gingiva, and floor of the mouth [2].

A torn labial frenum is widely regarded as pathognomonic of abuse [2]. However, most impalement injuries are accidental [5]. The most common mechanism of injury is a fall with an object in the mouth or hands [5]. Objects most commonly associated with impalement include toothbrushes, toys, sticks, writing instruments, and, in Asia, chopsticks [5]. The rare but serious consequences of oropharyngeal impalement include retropharyngeal and mediastinal abscesses, mediastinitis, widespread emphysema, internal carotid artery thrombosis and bleeding, and airway obstruction [5]. Since cases of abusive trauma rarely appear in the literature, a diagnostic approach and management strategy has not been fully elucidated. Unreported intraoral injuries may result from the clinician's lack of familiarity with the oral cavity or difficulty with effective examination of this area in young children [1]. In the case presented, multiple medical professionals cared for the patient without identifying the injury. Failure to recognize the injury may have occurred because the patient was uncooperative during physical examination and possibly in pain. Formal examination in the operating room was recommended due to the inability to fully examine this patient.

If earlier recognition had occurred, the patient could have possibly undergone primary palatal repair. Oral impalement injuries usually do not require closure [1]. The recommended management for simple puncture wounds is observation and follow-up care [1]. If the oral floor is involved, prophylactic antibiotics are given [1]. An abnormal neurovascular exam requires an angiogram [1]. When a complete oral examination proves to be difficult, the recommendation is an exam under anesthesia to determine whether repair is required [1]. The direct and thorough examination in the operating room can be critical in cases of trauma where the laceration may have been more forceful [1]. In this case, the injury appears to have been significant and extensive enough that primary closure at the time of injury would likely have been indicated. Failure to do so resulted in palatal and Eustachian tube dysfunction. By understanding oral and oropharyngeal anatomy, the clinician can better predict the natural history of an unexplained oral or oropharyngeal lesion and determine the proper management strategy or referrals.

Recognizing abuse relies not only on an instance of injury, but also on the context of a child's medical, social, and developmental history and the explanation offered for injury [2]. The granulation tissue indicative of wound healing on the patient's soft palate combined with the patient's high-risk age group suggests possible abuse. The social and developmental history revealed that the patient refused to eat, stopped growing, and lost weight. This arrest of development is a typical sequel abuse; however, dysphagia and odynophagia could also explain these findings. Finally, the history given by the mother did not explain the patient's presentation. The mother's denial of trauma leads us to believe the unexplained injury occurred while the child was under the care of the babysitter. Denial of trauma or a lack of explanation in cases of identified injuries should raise suspicion of child abuse. Attempts to screen for child abuse are hindered by the lack of a gold standard for detecting child abuse and low sensitivity of existing decision tools. Early detection proves crucial for managing consequences of the injury, preventing future instances of abuse, and decreasing morbidity and mortality. This case demonstrates the importance of early detection of traumatic injuries of the mouth and justifies recommending that intraoral hard and soft tissue be examined in all suspected abuse cases [2].

## 4. Conclusion

Complex cases of unexplained oral and oropharyngeal injuries may prove difficult to diagnose and manage, often requiring a consult with a dentist or otolaryngologist. However, early detection of oral injuries in children relies on all medical professionals' familiarity with the relevant anatomy and ability to examine it completely in uncooperative children. Advancing the knowledge of structural injuries in the context of a patient history can help elucidate tools for diagnosing abuse in the appropriate situations.
